# Supplementary figures and images for: vConTACT: an iVirus tool to classify double-stranded DNA viruses that infect Archaea and Bacteria
Source: PeerJ. 2017 May 3;5:e3243. doi: 10.7717/peerj.3243 (PMC5419219; doi:10.7717/peerj.3243)

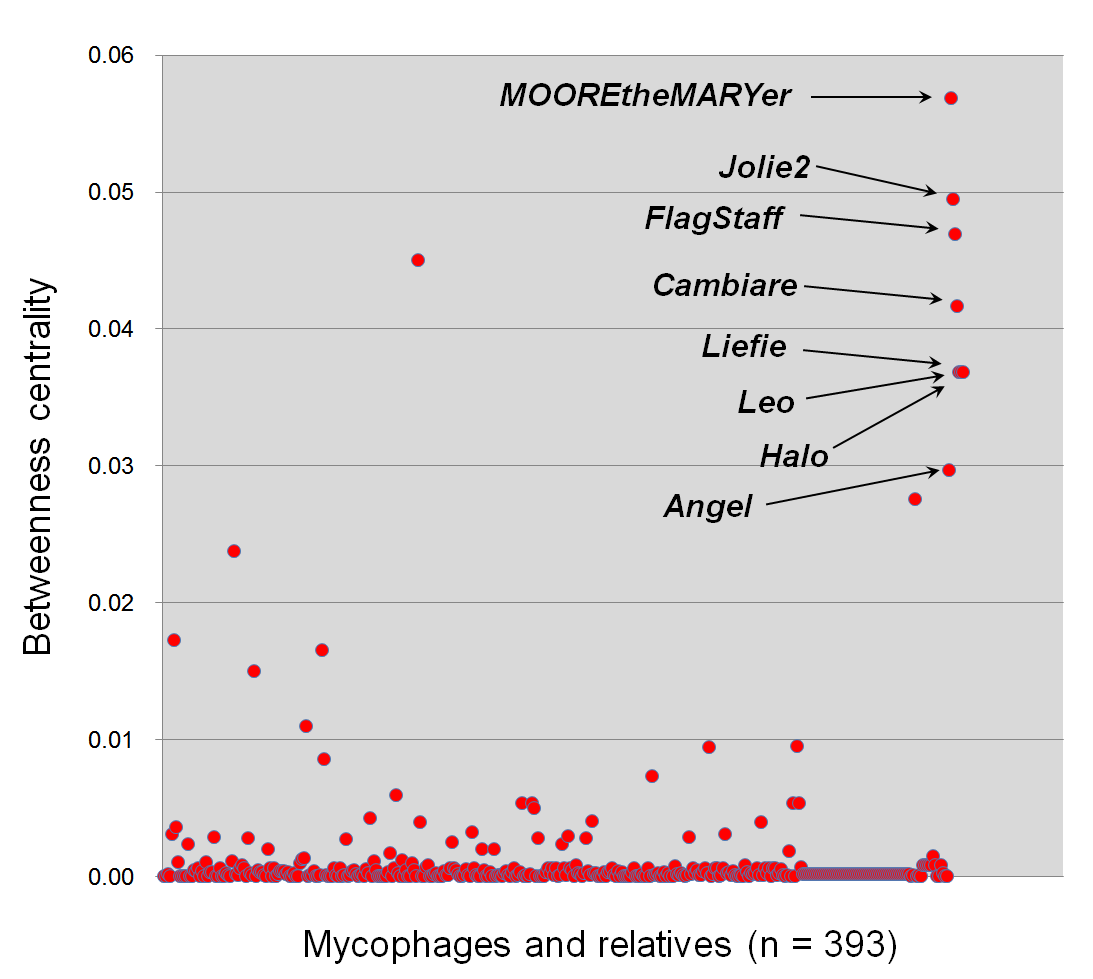

Supplement: Figure S2 — Each dot represents the betweenness centrality of an individual virus. The x-axis is the virus, and the y-axis is the value of betweenness centrality. A total of 393 mycophages and their relatives that link to mycophages are represented. Eight mycophages belonging to the VC59 are highlighted with arrows due to their higher betweenness values than other myco-/phages. Nodes with high betweenness possibly suggest that they play a central role in sharing of gene contents. [file peerj-05-3243-s006.png]

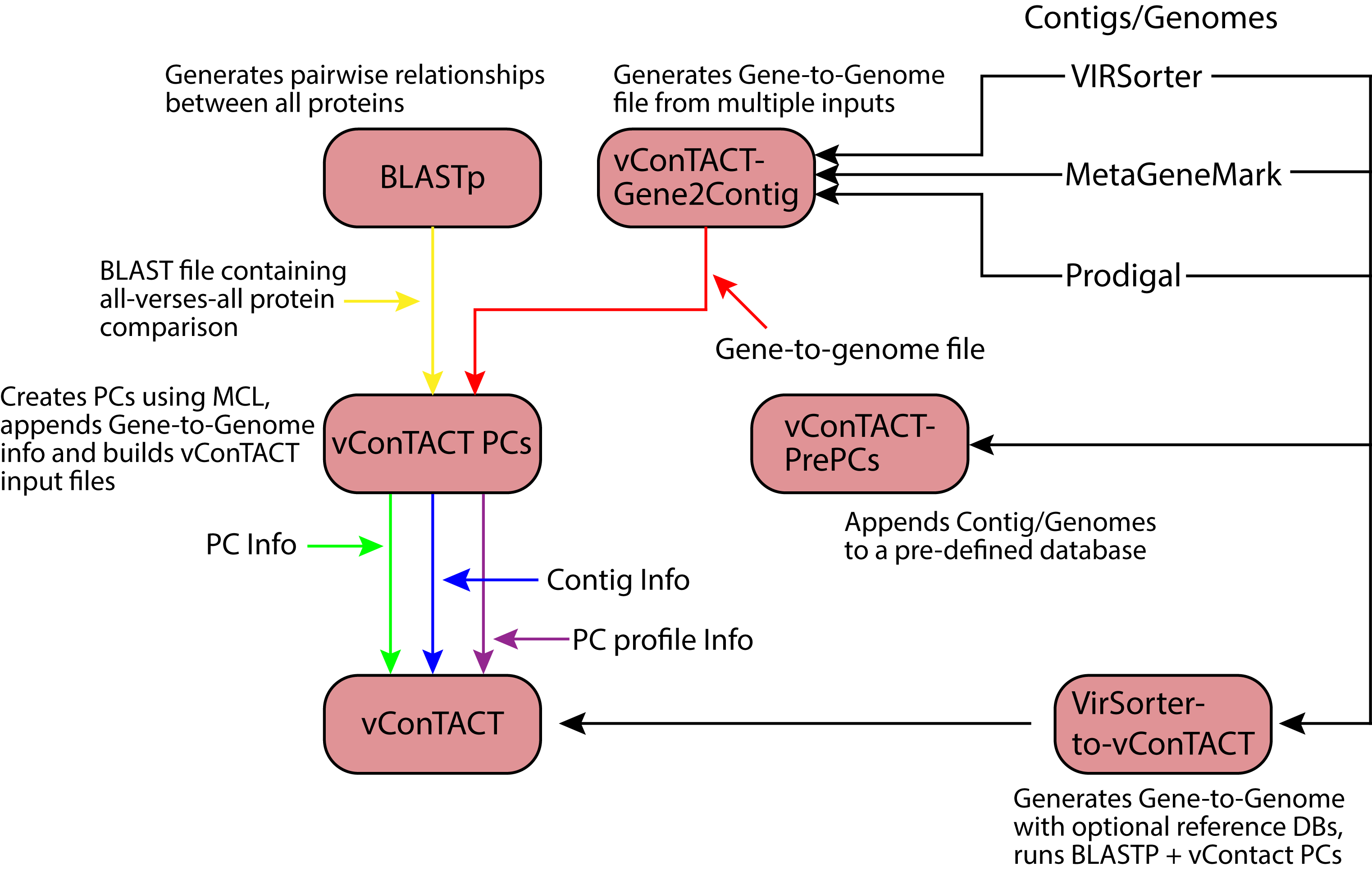

Supplement: Figure S5 [file peerj-05-3243-s009.png]
